# Technical Approach Determines Inflammatory Response after Surgical and Transcatheter Aortic Valve Replacement

**DOI:** 10.1371/journal.pone.0143089

**Published:** 2015-11-23

**Authors:** Gabor Erdoes, Christoph Lippuner, Istvan Kocsis, Marcel Schiff, Monika Stucki, Thierry Carrel, Stephan Windecker, Balthasar Eberle, Frank Stueber, Malte Book

**Affiliations:** 1 Department of Anesthesiology and Pain Medicine, Inselspital, Bern University Hospital, University of Bern, Bern, Switzerland; 2 Department of Clinical Research, Anesthesiology Group, Inselspital, Bern University Hospital, University of Bern, Bern, Switzerland; 3 2^nd^ Department of Obstetrics and Gynecology, Semmelweis University of Medicine, Budapest, Hungary; 4 Department of Cardiovascular Surgery, Swiss Cardiovascular Center, Inselspital, Bern University Hospital, University of Bern, Bern, Switzerland; 5 Department of Cardiology, Swiss Cardiovascular Center, Inselspital, Bern University Hospital, University of Bern, Bern, Switzerland; University of Colorado Denver, UNITED STATES

## Abstract

**Objective:**

To investigate the periprocedural inflammatory response in patients with isolated aortic valve stenosis undergoing surgical aortic valve replacement (SAVR) or transcatheter aortic valve implantation (TAVI) with different technical approaches.

**Material and Methods:**

Patients were prospectively allocated to one of the following treatments: SAVR using conventional extracorporeal circulation (CECC, n = 47) or minimized extracorporeal circulation (MECC, n = 15), or TAVI using either transapical (TA, n = 15) or transfemoral (TF, n = 24) access. Exclusion criteria included infection, pre-procedural immunosuppressive or antibiotic drug therapy and emergency indications. We investigated interleukin (IL)-6, IL-8, IL-10, human leukocyte antigen (HLA-DR), white blood cell count, high-sensitivity C-reactive protein (hs-CRP) and soluble L-selectin (sCD62L) levels before the procedure and at 4, 24, and 48 h after aortic valve replacement. Data are presented for group interaction (p-values for inter-group comparison) as determined by the Greenhouse-Geisser correction.

**Results:**

SAVR on CECC was associated with the highest levels of IL-8 and hs-CRP (p<0.017, and 0.007, respectively). SAVR on MECC showed the highest descent in levels of HLA-DR and sCD62L (both p<0.001) in the perioperative period. TA-TAVI showed increased intraprocedural concentration and the highest peak of IL-6 (p = 0.017). Significantly smaller changes in the inflammatory markers were observed in TF-TAVI.

**Conclusion:**

Surgical and interventional approaches to aortic valve replacement result in inflammatory modulation which differs according to the invasiveness of the procedure. As expected, extracorporeal circulation is associated with the most marked pro-inflammatory activation, whereas TF-TAVI emerges as the approach with the most attenuated inflammatory response. Factors such as the pre-treatment patient condition and the extent of myocardial injury also significantly affect inflammatory biomarker patterns. Accordingly, TA-TAVI is to be classified not as an interventional but a true surgical procedure, with inflammatory biomarker profiles comparable to those found after SAVR. Our study could not establish an obvious link between the extent of the periprocedural inflammatory response and clinical outcome parameters.

## Introduction

The best valve replacement technique for patients with symptomatic severe aortic stenosis (AS) is currently under discussion, particularly as robust evidence suggests that surgical aortic valve replacement (SAVR) and transcatheter aortic valve implantation (TAVI) show comparable clinical outcomes in patients at high surgical risk due to severe co-morbidities [1,2]. Currently, the decision about the most appropriate approach is made by a multidisciplinary Heart Team, based on overall life expectancy, coexisting diseases, anatomical conditions and frailty.

Recent research indicates that plasma biomarker profiles may be predictive of long-term success of aortic valve replacement. NT-proBNP, carbohydrate antigen CA125 and osteoprotegerin were found to be associated with cardiovascular mortality and death if remained high after the procedure [3–5]. Similarly, the inflammatory state and extent of the inflammatory response may be indicative of the patients’ clinical course. In this regard, elevated levels of high-sensitivity C-reactive protein (hs-CRP) or white blood cells (WBC), as part of a systemic inflammatory response syndrome (SIRS), have been shown to predict outcome and mortality, particularly in the presence of diabetes [6–8]. The procedural pro-inflammatory state may be further characterized by the measurement of cytokines, a broad category of small proteins involved in cell signaling processes. The release and time course of the cytokine-mediated inflammatory response have been investigated in patients with coronary artery disease (CAD) undergoing coronary artery bypass grafting with and without the use of extracorporeal circulation (ECC) [9]. However, these results cannot be simply translated to patients treated for AS because of the different pathogenesis and molecular mechanisms involved in coronary versus valvular degenerative disease. In severe AS, the observed pathological processes suggest that inflammation, known to play a key role in atherosclerosis, is diminished, whereas osteogenic, metabolic and endocrine mechanisms predominate [10,11]. In fact, the progression of AS is primarily driven by osteoblast activity, resulting in progressive leaflet calcification, in contrast to atherosclerosis, where inflammation and lipid deposition are the key components [12]. Due to further disparities with regard to the clinical and treatment characteristics involved in the release of cytokines (e.g., increased left ventricular myocardial mass in patients with AS,[13] the placement of aortic prosthesis as a foreign material), the inflammatory response is only partially comparable and may not lead to the same conclusions in CAD and AS treatment strategies.

Technical innovations in the field of cardiopulmonary bypass technology now allow for aortic valve replacement using minimized (MECC) instead of conventional extracorporeal circuits (CECC). MECC has also been shown to result in less pronounced activation of the innate immune system, due to reduced blood-air contact, modified cardiotomy suction and use of biocompatible coating in the circuit [14,15]. A further reduction of cytokine response has been reported with use of transapical TAVI (TA-TAVI) as compared to SAVR using CECC [16].

The aim of this study was to investigate the periprocedural course of a common selection of pro- and anti-inflammatory markers in patients with AS, using all currently available aortic valve replacement techniques at a single institution. The hypothesis was that the most severe impairment of the immune system occurs with SAVR on CECC, followed by SAVR on MECC and TA-TAVI, which are associated with a smaller trauma. We expected only minimal activation of the immune response with transfemoral TAVI (TF-TAVI) due to the avoidance of extra-corporeal circulation, surgical approach and direct myocardial injury.

## Materials and Methods

### Study participants

All patients with symptomatic severe aortic stenosis, defined as an aortic valve area <1 cm^2^ and an aortic valve mean pressure gradient >40 mmHg, scheduled for either surgical or transcatheter treatment at our institution, were prospectively assessed for eligibility between September 2010 and April 2013. Exclusion criteria included clinically or laboratory apparent local or systemic infection, chronic intake of immunosuppressive drugs or antibiotics and emergency indications. All included individuals provided written informed consent for participation in the study. Approval (reference number: 041/09) was granted by the Cantonal Ethics Committee (Kantonale Ethikkommission Bern, Postfach 56, 3010 Bern, Switzerland, Contact: Dr. sc. nat. Dorothy Pfiffner). The study was registered with Clinicaltrials.gov (NCT02324140) and was performed in compliance with the Declaration of Helsinki and the STROBE criteria [17].

### Management of aortic valve stenosis

Treatment selection was based on the patients’ clinical, echocardiography and computed tomography findings, and was decided upon by the institutional Heart Team, consisting of cardiac surgeons and interventional cardiologists. Patients were considered for transcatheter treatment if they were older than >80 years and/or a logistic EuroScore predicted risk of mortality ≥15%, a Society of Thoracic Surgeons’ Score risk of mortality ≥10%, or age >70 years with a predicted or prohibitive risk of morbidity or/and mortality for SAVR. In all other cases, SAVR was performed using CECC. MECC was used in a subset of SAVR patients. Patients selected for transcatheter treatment received TA-TAVI or TF-TAVI. Access route was selected by the Heart Team and thus, not randomly.

### Surgical aortic valve replacement and extracorporeal perfusion

The MECC system (Jostra AG, Hirrlingen, Germany) consisted of a centrifugal pump (Rotaflow, Maquet, Gossau, Switzerland), a microporous capillary membrane oxygenator (Quadrox; Maquet, Gossau, Switzerland or Capiox FX25; Terumo, Tokyo, Japan), an anesthesia vaporizer (isoflurane), a pulmonary artery vent line and an optoelectrically activated suction device (SmartSuction, Cardiosmart, Muri, Switzerland). CECC included a roller pump (Maquet, Rastatt, Germany), a hardshell cardiotomy reservoir, conventional cardiotomy suction, and a left ventricular vent line inserted via the right upper pulmonary vein pulmonary vein and connected to the cannula of venous return. MECC was primed with 600 mL of Ringer’s solution and 5,000 IU of heparin; CECC was primed with 500 mL of hydroxyethyl starch (130/0.4), 1,000 mL of Ringer’s solution, 100 mL of 20% mannitol and 10,000 IU of heparin. None of the extracorporeal circuits used heparin coating or neutrophil filter.

General anesthesia was induced with midazolam, etomidate and sufentanil, followed by isoflurane and sufentanil maintenance. Surgery was performed through a median sternotomy. After heparinization (MECC, 400 IU/kg body weight; CECC, 500 IU/kg), extracorporeal circulation was established, with arterial inflow through the ascending aorta and venous drainage through a two-stage cannula secured in the right atrium. An activated clotting time (ACT_kaolin_) of >480 s (ACT Plus, Medtronic Ltd., MN, USA) was targeted in both groups and monitored every 20 min. All patients received a bolus of tranexamic acid (30 mg/kg) followed by a continuous infusion (15 mg/kg/h) until sternal closure. Extracorporeal circuit flow rates were set to 2.0 L/min/m^2^ body surface area (BSA) in MECC, and 2.4 L/min/m^2^ BSA in CECC. Core temperature was allowed to drift to a nadir of 34°C. Myocardial protection was applied with a single dose of 100 mL of crystalloid cardioplegia (Cardioplexol, Bichsel Laboratory, Interlaken, Switzerland), followed by high-potassium cold blood cardioplegia, with repetition every 20–30 minutes or if any electrical or mechanical activity was observed. Following weaning from cardiopulmonary bypass, heparin was neutralized with protamine sulfate in a ratio of 1:1 with regard to the initial bolus.

### Transcatheter aortic valve implantation

TA-TAVI and TF-TAVI were performed as described previously by our group and others [18,19]. In all TA-TAVI cases, the balloon-expandable Edwards Sapien XT aortic bioprosthesis (Edwards Lifesciences Inc., Irvine, CA, USA) was used and deployed by a single implantation step under rapid ventricular pacing. For TF-TAVI, either the Edwards Sapien XT aortic bioprosthesis or the self-expanding CoreValve Revalving System (Medtronic Inc., Minneapolis, MN, USA) was used. All procedures were performed following balloon valvuloplasty of the native calcified aortic valve.

The periprocedural antithrombotic regimen consisted of a bolus of heparin (70–100 IU/kg), which was repeated with half of the initial dose if ACT_kaolin_ remained below 250 s. Postprocedurally, all TAVI patients received acetylsalicylic acid (100 mg) and a loading dose of clopidogrel (300 mg).

TA-TAVI patients received general endotracheal anesthesia as described for SAVR. TF-TAVI was routinely performed using local anesthesia combined with dexmedetomidine sedation.

All patients received a bolus of cefuroxime (SAVR: 1.5 g/kg; TAVI 750 mg/kg) 30–40 min prior to the skin incision and at the end of the procedure. Postoperatively and under continued general anesthesia, all SAVR and TA-TAVI patients were transferred to the intensive care unit, whereas the TF-TAVI patients were extubated and transferred to the cardiology intermediate care (IMC) unit.

### Blood sampling and determination of inflammatory resonse markers

In all cases, blood was collected in three different tubes (2x EDTA tubes with a filling volume of 2.7 mL and 1x heparinized tube with a filling volume of 4.7 mL), which were immediately transferred to the in-house laboratory. The first sample (baseline value) was drawn from the patient`s arterial line prior to administration of anesthetic drugs. Subsequent samples were drawn from the central venous line after 4, 24 and 48 h post-operatively.

### HLA-DR expression

Fifty microliters of heparinized blood were stained with 20 μL of Quantibrite^™^ anti-human-HLA-DR PE (phycoerythrin)/anti-monocyte PerCP (peridinin chlorophyll protein)-Cy5.5 (anti-human-CD14 and anti-human-CD64) (BD Biosciences) at room temperature in the dark for 25 min. Red blood cells were subsequently lysed with FACS lysis solution (BD Biosciences) for 5–10 min, washed twice with phosphate-buffered saline solution (Sigma-Aldrich) and fixed with 400 μL of 4% paraformaldehyde. One unstained sample was treated in the same manner. The fluorescence intensity of the samples was measured in duplicate on an LSR II (BD Biosciences) using the software FACSDiva v6.1.3 (BD Biosciences). A total of 500–1,000 monocyte events were recorded. For quantification of the signal, Quantibrite^™^ PE beads (BD Biosciences) were acquired with each measurement. The FACS data were analyzed using FlowJo v7.5 (Treestar) with gating for CD14- and CD64-positive cells (monocytes). The HLA-DR PE channel was calibrated using the data from the PE beads, which allows fluorescence intensity to be correlated with the mean number of PE molecules per cell. Results were recorded as the median of the calibrated PE channel fluorescence intensity of each sample. The mean and standard deviation were calculated for the duplicate samples, and an analysis of variance (ANOVA) was performed using GraphPad Prism v5.04 (GraphPad Software).

### ELISA

Plasma from each time point was separated from 5 mL of EDTA-whole blood by centrifugation at 3,000 g for 5 min and stored at -80°C.

### IL-6 ELISA

Interleukin-6 levels were measured by a sandwich ELISA kit (eBioScience), and all samples were diluted 1:2 using the reagents provided by the kit. The standards ranged from 1.56 to 100 pg/mL. The provided 96-well plate was treated according to the manufacturer’s instructions, and all standards, controls and samples were loaded in duplicate. Optical density was measured using an eL800 microplate reader (Biotek Instruments) set to record at 450 and 630 nm. Blank values and OD values at 630 nm were subtracted from all OD 450 nm values, and the IL-6 concentration of all samples was determined by a 4-parameter curve fit of the standards (Gen5 v.1.09 software, Biotek Instruments). Samples that fell out of the standard curve range were repeated at a higher dilution. According to the manufacturer, the sensitivity and intra- and inter-assay variations were 0.92 pg/mL, 3.4% and 5.2%, respectively. A coefficient of variation of the sample duplicates of below 20% was considered acceptable.

### IL-8 ELISA

Interleukin-8 levels were measured by a sandwich ELISA kit (eBioScience), and all samples were diluted 1:2 using the reagents provided by the kit. The standards ranged from 15.6 pg/ml to 1000 pg/ml. The provided 96-well plate was treated according to the manufacturer’s instructions and all standards, controls and samples were loaded in duplicate. Optical density was measured using an eL800 microplate reader (Biotek Instruments) set to record at 450 and 630 nm. Blank values and OD values at 630 nm were subtracted from all OD 450 nm values and the IL-8 concentration of all samples was determined by a 4-parameter curve fit of the standards (Gen5 v.1.09 software, Biotek Instruments). Samples that fell out of the standard curve range were repeated at a higher dilution. According to the manufacturer, the sensitivity and the intra- and inter-assay variations were 2 pg/ml, 6.3% and 8.7%, respectively. A coefficient of variation of the sample duplicates of below 20% was considered acceptable.

### IL-10 ELISA

Interleukin-10 levels were measured by a sandwich ELISA kit (eBioScience), and all samples were diluted 1:2 using the reagents provided by the kit. The standards ranged from 3.1 to 200 pg/mL. The provided 96-well plate was treated according to the manufacturer’s instructions, and all standards, controls and samples were loaded in duplicate. Optical density was measured using an eL800 microplate reader (Biotek Instruments) set to record at 450 and 630 nm. Blank values and OD values at 630 nm were subtracted from all OD 450 nm values, and the IL-10 concentration of all samples was determined by a 4-parameter curve fit of the standards (Gen5 v.1.09 software, Biotek Instruments). Samples that fell out of the standard curve range were repeated at a higher dilution. According to the manufacturer, the sensitivity and intra- and inter-assay variations were 1.0 pg/mL, 3.2% and 5.6%, respectively. A coefficient of variation of the sample duplicates of below 20% was considered acceptable.

### sCD62L

sL-selectin (sCD62L) levels were measured by a sandwich ELISA kit (eBioScience), and all samples were diluted 1:200 using the reagents provided by the kit. The standards ranged from 0.4 to 25 ng/mL. The provided 96-well plate was treated according to the manufacturer’s instructions, and all standards, controls and samples were loaded in duplicate. Optical density was measured using an eL800 microplate reader (Biotek Instruments) set to record at 450 and 630 nm. Blank values and OD values at 630 nm were subtracted from all OD 450 nm values, and the sCD62L-selectin concentration of all samples was determined by a 4-parameter curve fit of the standards (Gen5 v.1.09 software, Biotek Instruments). Samples that fell out of the standard curve range were repeated at a higher dilution. According to the manufacturer, the sensitivity and intra- and inter-assay variations were 0.198 pg/mL, 3.7% and 4.2%, respectively. A coefficient of variation of the sample duplicates of below 20% was considered acceptable.

### hs-CRP

High-sensitivity C-reactive protein levels (hs-CRP) were measured by an ELISA kit (eBioScience), and all samples were diluted 1:8,000, 1:16,000, 1:32,000, 1:40,000 or 1:64,000 using the reagents provided by the kit. The dilution was determined by several pre-tests. The standards ranged from 0.15 to 10 ng/mL. The provided 96-well plate was treated according to the manufacturer’s instructions, and all standards, controls and samples were loaded in duplicate. Optical density was measured using an eL800 microplate reader (Biotek Instruments) set to record at 450 and 560 nm. Blank values and OD values at 560 nm were subtracted from all OD 450 nm values, and the IL-6 concentration of all samples was determined by a 4-parameter curve fit of the standards (Gen5 v.1.09 software, Biotek Instruments). Samples that fell out of the standard curve range were repeated at a higher dilution. A coefficient of variation of the sample duplicates of below 20% was considered acceptable.

### Statistical analysis

Statistical data were analyzed in IBM Statistics SPSS v22. A general linear model for repeated measures followed by the Bonferroni correction was used to compare the different procedures. P values <0.05 were considered significant. The Greenhouse-Geisser correction was applied to inter-subject effects. Time-dependent changes in cytokine levels were analyzed by the Friedman test. To examine where the differences actually occurred, the Wilcoxon signed-rank test was performed on the different combinations of related groups. Because of these multiple comparisons Bonferroni adjustment of the Wilcoxon test results was performed, i.e., for four comparisons, p values <0.0125 (p = 0.05 or less; 0.05/4) were considered significant.

## Results

### General data

During the study period, 316 patients underwent isolated SAVR and 402 patients TAVI at our institution. Considering the exclusion criteria and exclusions due to logistical reasons, 101 patients (SAVR on CECC: 47, SAVR on MECC: 15, TA-TAVI: 15, TF-TAVI: 24) were included in the final analysis and provided complete inflammatory datasets within the study period. As expected, the groups’ preoperative characteristics differed significantly with regard to age, NYHA class, incidence of coronary artery disease, chronic obstructive pulmonary disease and previous (redo) cardiothoracic surgery. The patients selected for SAVR on MECC were the youngest (mean age 65 years, p <0.001) and had the lowest number of co-morbidities. Procedural time was the shortest in patients undergoing TF-TAVI (75 min, p <0.001). TF-TAVI patients had the longest monitored care (48 h on the IMC unit vs. 20–22 h ICU stay in other groups, p <0.001). Post-operative hospital length of stay (post-op LOS), defined as the number of days between treatment for aortic stenosis and hospital discharge, was significantly different (p = 0.04) having a median of 9 days in TA-TAVI recipients, 7 days in TF-TAVI and 8 days in surgical patients, respectively.

SIRS, as defined by the American College of Chest Physicians/Society of Critical Care Medicine,[20] developed at a significantly higher rate in the TA-TAVI group than in the other groups (4/15 pts., p = 0.02). One patient died in the TA-TAVI group. No significant differences regarding new infection (p = 0.80), incidence of stroke (p = 0.71) and 30-day mortality (p = 0.13) were observed between the surgical and transcatheter treatment groups (Table 1).

**Table 1:** Patients`and procedural characteristics.

	TF-TAVI (n = 24)	TA-TAVI (n = 15)	MECC (n = 15)	CECC (n = 47)	p
Baseline characteristics					
Age,yr.	82±4	79 ±5	65±8	68±9	<0.001*
Female sex,n	9 (37)	5 (33)	3 (20)	15 (32)	0.13
BMI,kg/m^2^	28 ±4	24 ± 4	28 ± 5	28 ± 5	0.14
NYHA class,n					
I	0 (0)	1(7)	1(7)	5(11)	0.43
II	5 (21)	3(20)	9(60)	23(49)	0.02*
III	15 (62)	10(66)	4(26)	15(32)	0.01*
IV	4 (17)	1(7)	1(7)	4(8)	0.72
CAD,n	14(58)	8(53)	1(7)	7(15)	<0.001*
Diabetes,n	7(29)	6(40)	3(20)	9(19)	0.31
Statins,n	6(25)	8(53)	7(47)	17(36)	0.29
COPD,n	6(25)	4(27)	0(0)	0(0)	<0.001*
Dialysis,n	0(0)	1(6)	1(7)	1(2)	0.52
Redo surgery,n	10(42)	4(27)	1(7)	2(4)	<0.001*
Ejection fraction,%	60 [45;65]	55 [40;65]	60 [55;70]	60 [59;65]	0.14
Aortic valve area,cm^2^	0.7[0.6;0.8]	0.7[0.4;0.4]	0.7 [0.5;0.8]	0.7 [0.5;0.8]	0.77
Δp mean,mmHg	41 [29;57]	34 [27;59]	47 [40;57]	42 [34;59]	0.59
Procedural characteristics					
Procedural time,min	75[58;89]	100[84;114]	152[130;173]	165[145;185]	<0.001*
ECC time,min			65[51;74]	66[56;86]	0.28
Cross clamp time,min			49[42;55]	47[40;64]	0.87
Outcome					
IMC / ICU time,h	48[33;75]	20[17;23]	21[18;22]	22[21;23]	<0.001*
Post-op LOS,d	7[6;11]	9[9;11]	8[7;9]	8[7;10]	0.04*
New infection,n	1(4)	1(7)	1(7)	1(2)	0.80
SIRS^a^,n	0(0)	4(27)	1(7)	3(6)	0.02*
Stroke,n	1(4)	0(0)	1(7)	1(2)	0.71
30-d mortality,n	0(0)	1(7)	0(0)	0(0)	0.13

TF-TAVI indicates transcatheter transfemoral aortic valve replacement; TA-TAVI, transcatheter transapical aortic valve replacement; MECC, minimized extracorporeal circulation; CECC, conventional extracorporeal circulation; BMI, body mass index; NYHA, New York Heart Association class; CAD, coronary artery disease; COPD, chronic obstructive pulmonary disease; Δp mean, mean transaortic gradient; ECC, extracorporeal circulation; IMC, intermediate care unit; ICU, intensive care unit; Post-op LOS, post-operative hospital length of stay; SIRS, systemic inflammatory response syndrome.

^a^Definition according to guidelines [20].

Values are number (percent), mean ± standard deviation or median with interquartile range. P <0.05 is considered significant.

### Cytokine release

**Fig 1:**
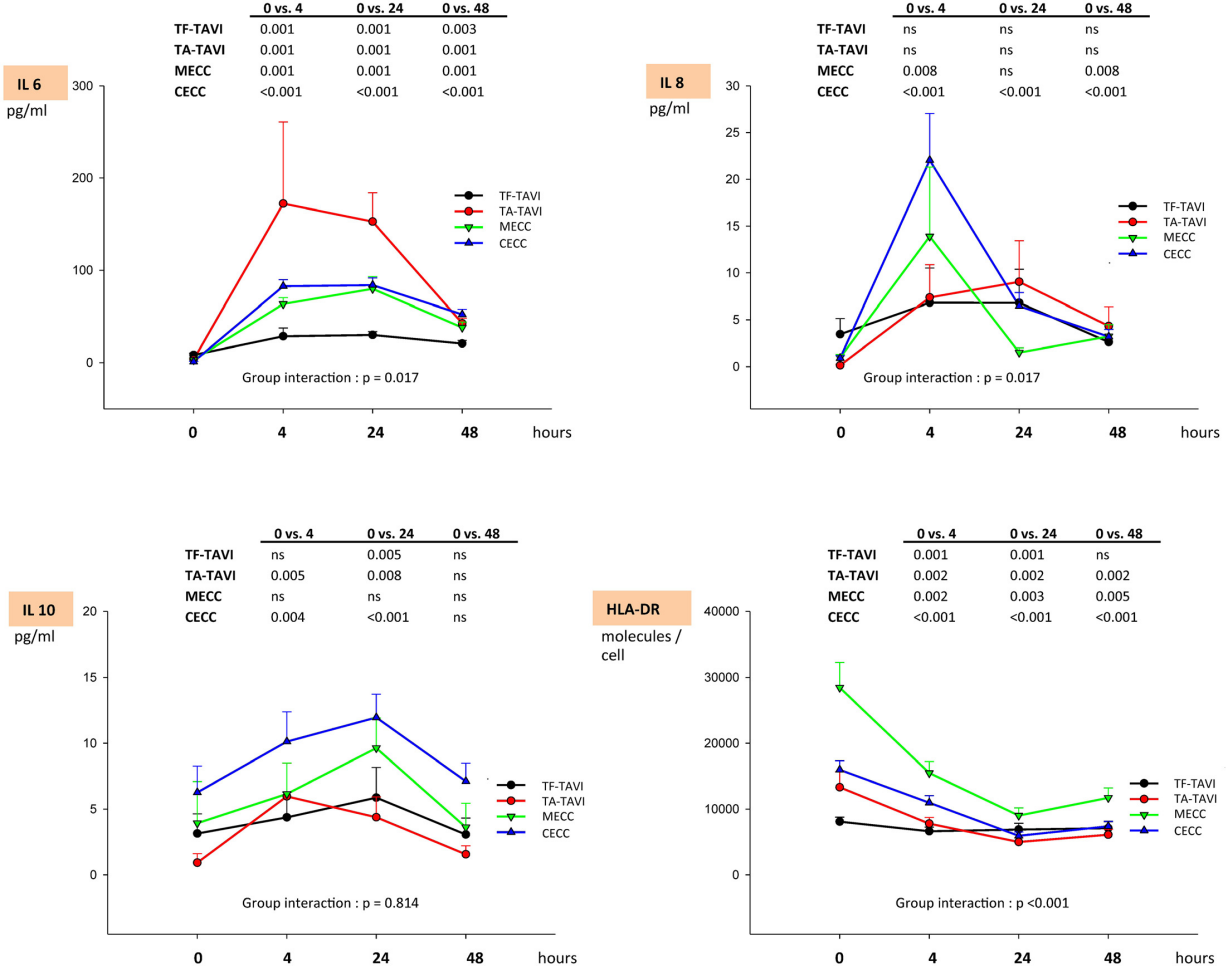
Biochemical markers of inflammatory response I. The table indicates p-values as a result of comparisons within different time points within a treatment group as determined by Wilcoxon test with Bonferroni adjustment. Group interaction indicates p values for intra-subject effects as determined by Greenhouse-Geisser (ANOVA on ranks with Bonferroni adjustment). Data are expressed as mean ± standard error of mean (SEM). Legend: TF-TAVI indicates transcatheter transfemoral aortic valve replacement; TA-TAVI, transcatheter transapical aortic valve replacement; MECC, minimized extracorporeal circulation; CECC, conventional extracorporeal circulation.

TF-TAVI patients showed the lowest plasma level of IL-6 and IL-8 and a significantly different course from the other groups (p = 0.017, Fig 1). The highest levels of pro-inflammatory cytokines were observed in patients undergoing TA-TAVI for IL-6, and in CECC for IL-8. IL-6 peaked after 4 h and remained significantly above baseline after 48 h in all groups (all p ≤0.001). Similarly, IL-8 peaked after 4 h except in TA-TAVI and TF-TAVI patients. In contrast to TF-TAVI and TA-TAVI patients where IL-8 was at baseline levels after 48 h (p >0.05), the IL-8 plasma level of MECC and CECC patients were still elevated at 48 h (MECC: p = 0.008; CECC: p <0.001). The IL-10 plasma level did not differ between the groups (p = 0.814, Fig 1). IL-10 peaked at 24 h in CECC, MECC and TF-TAVI, but at 4 h already in TA-TAVI. In all groups, after 48 h IL-10 was not significantly different from baseline.

### Human leucocyte antigen expression

The highest human leukocyte antigen (HLA)-DR level on circulating monocytes was detected in MECC, followed by CECC and TA-TAVI (group interaction: p = 0.001, Fig 1). With the exception of the TF-TAVI group, patients allocated to other treatment options exhibited a significantly reduced level of HLA-DR after 48 h when compared to baseline (p <0.005).

### White blood cell count and high-intensity C-reactive protein levels

**Fig 2:**
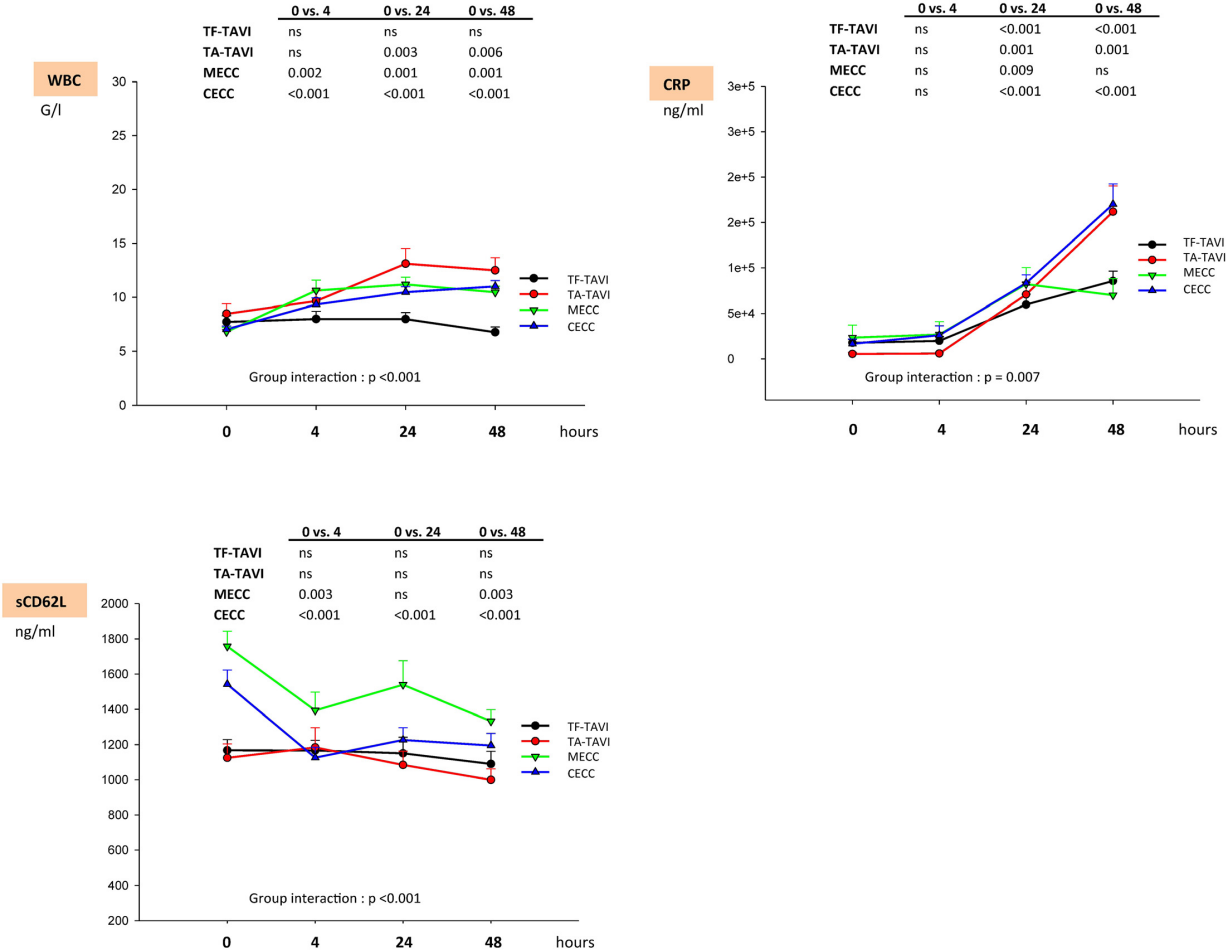
Biochemical markers of inflammatory response II. The table indicates p-values as a result of comparisons within different time points as determined by Wilcoxon test with Bonferroni adjustment. Group interaction indicates p values for inner-subject effects as determined by Greenhouse-Geisser (ANOVA on ranks with Bonferroni adjustment). Data are expressed as mean ± standard error of mean (SEM). Legend: TF-TAVI indicates transcatheter transfemoral aortic valve replacement; TA-TAVI, transcatheter transapical aortic valve replacement; MECC, minimized extracorporeal circulation; CECC, conventional extracorporeal circulation; WBC, white blood cells; CRP, high-sensitivity C-reactive protein; sCD62L, soluble CD62L.

White blood cell count (WBC) and hs-CRP levels showed different courses within the groups (group interaction: p <0.001, Fig 2). The WBC count increased to a maximum at 24 h in all groups but returned to nearly the same level after 48 h. Only TF-TAVI patients showed no significant elevation of WBC count for all time points. With all other treatment options, the WBC levels remained increased at 48 h (p <0.006). Hs-CRP levels peaked significantly in all groups during the observation period with the exception of the MECC group, which exhibited a non-significant difference from baseline at 48 h.

### Soluble CD62L levels

Soluble CD62L (sCD62L) was the highest in the MECC group and lowest in TA-TAVI, showing significantly different courses in the first 48 h postoperatively (p <0.001, Fig 2). With the exception of the TAVR patients, sCD62L levels in the MECC and CECC groups differed significantly from baseline (p <0.003).

## Discussion

We analyzed the periprocedural inflammatory response of patients with symptomatic severe aortic stenosis who underwent either surgical or transcatheter aortic valve replacement. Blood samples were collected before and at 4, 24 und 48 h after SAVR using CECC or MECC, TA-TAVI or TF-TAVI, respectively and were analyzed for selected cytokine levels, HLA-DR, WBC, hs-CRP, and sCD62L.

Comparing the different treatment modalities, the periprocedural inflammatory response differed significantly depending on the type of the procedure. As expected, the inflammatory response was most attenuated in TF-TAVI and most pronounced with CECC. However, the kinetics and time-course of the investigated inflammatory markers could not be explained entirely by the presence or absence of an extracorporeal circuit and its attributed activation of inflammatory pathways.

In accordance with other reports, the high levels of IL-8 and IL-10 reflected the contribution of the conventional extracorporeal circuit to the inflammatory and associated anti-inflammatory responses [9,15,16,21,22]. Activation of circulating leukocytes by CECC is likely due to the larger artificial surface area, the blood-air contact in the open hardshell venous reservoir and the cardiotomy suction [23–27]. The inflammatory response was considerably reduced when SAVR was performed with a MECC system, which has shorter tubing and lower priming volume. Lesser degrees of hemodilution, mechanical stress or blood activation by contact with air with less release of IL-6, IL-8, IL-10 and hs-CRP confirm modulation of the inflammatory cascade by the properties of the extracorporeal circuit [14,15,28,29].

IL-6 as another marker of an inflammatory state, showed the highest levels after TA-TAVI. IL-6 is a pro-inflammatory cytokine comparable to IL-8 increasing with ischemia/reperfusion injury. The latter causes thrombin deposition and subsequent cytokine release under cardiopulmonary bypass conditions [14,30,31]. IL-6 release appears to be closely associated with tissue trauma [32,33]. Cardiac myocytes are the major source of IL-6 synthesis [13,34]. TA-TAVI, performed via anterolateral thoracotomy at normal temperatures and transapical access with transmyocardial purse-string sutures seems to be associated with more myocardial cell trauma than CECC or MECC, where no direct myocardial trauma occurs (no myocardial incision needed for valve replacement) and the myocardium is protected by mild body hypothermia and cold cardioplegic arrest. The substantial incidence of SIRS in the TA-TAVI population could be related to increased IL-6 secretion but also to unilateral lung ventilation during part of the procedure [7]. This condition, together with a longer procedural interval and a relatively high incidence of SIRS in the TA-TAVI population, may be responsible for the conflicting results in a similar study comparing TA-TAVI with SAVR using CECC [16]. IL-6 levels did not return to baseline in our treatment groups, which may indicate that post-procedural IL-6 release continues beyond 48 h [31].

An inflammatory reaction based on non-pathogen induced activation often shows reduced expression of HLA-DR on monocytes, accompanied by an impaired ability to present foreign antigen to immune cells (antigene presentation) in response to surgical trauma [35]. In our cohort, MECC patients were the youngest and had the lowest incidence of significant comorbidities. Their corresponding HLA-DR levels were high, indicating negligible baseline immunosuppression. In contrast, TF-TAVI patients appeared already immunosuppressed before aortic valve implantation. The transcatheter procedure in these patients did not lead to further immunomodulation. This is consistent with previous studies suggesting a reduced inflammatory stimulus with the transfemoral access route [36,37]. Interestingly, CECC and TA-TAVI affected the HLA-DR course similarly. In both groups, HLA-DR drifted to a nadir at 24 h, whereby the levels after 48 h were still significantly reduced when compared to baseline. With a lack of comparable data, it may only be hypothesized that irrespective of the technique used for aortic valve replacement, the intraoperative monocyte function is similarly compromised in CECC and TA-TAVI. In CECC, the use of an extracorporeal circuit and in TA-TAVI the surgical trauma may be responsible. Nevertheless, impact on the HLA-DR course was less since there was an already low baseline level of HLA-DR. The largest HLA-DR decline was seen in the MECC group. An increased susceptibility for infection in MECC appears less likely than a better pre-served preoperative immune state.

Low levels of sCD62L have been shown to indicate reduced CD62L shedding, suggestive of alleviated neutrophil activity [38–40]. Our data suggest that extracorporeal techniques impair neutrophil activity more than transcatheter techniques. Nevertheless, sCD62L may serve as a marker of endothelial lesion and neutrophil activation which are not detectable by conventional laboratory analysis. The extent of endothelial damage and inflammatory response induced by CECC may indeed be less pronounced than indicated by cytokines [9].

Our study has several limitations. The small sample size per group of treatment, the lack of randomization and the single-center design. Also, we measured inflammatory response only via circulating biomarker levels but not by gene expression in organ biopsies. Therefore, our conclusions refer only to the selected inflammatory marker profiles and not to gene expression in organ biopsies. Nevertheless, our selection of IL-6, IL-8, IL-10, hs-CRP and WBC corresponds to other studies and allows comparison. Also, the HLA-DR / monocyte kit chosen limits our analysis to activated monocytes only. Study of the entire monocyte population (only CD14+ without using the CD64 marker) might have yielded a wider view of HLA-DR status. In addition, the high-risk cohort of patients of our study has multiple comorbidities and concurrent medications with heterogeneous effects on the inflammatory response. Another important point is the fact that a majority of our patients were on statins and acetylsalicylic acid, which are known to have anti-inflammatory properties. Thus, the periprocedural course of inflammatory markers was necessarily modified to some degree. Finally, in cardiac surgery a wide range of perfusion techniques is used and the presented results corresponds to an institutional cardiopulmonary bypass strategy. The observed differences in pro-inflammatory marker profiles may therefore differ with, e.g., different oxygenators, surface coating or use of neutrophil filtering. Finally, as IMC/ICU stay and LOS are also dependent on logistic reasons (e.g., institutional guidelines for post-treatment monitoring time frame of conduction disorders, extent of physiotherapeutic measures, waiting time for medical rehabilitation services), these variables should be interpreted in conjuction with national characteristics of healthcare and not as a direct consequence of the patient`s healthcare condition or the selected treatment strategy for aortic stenosis itself.

In conclusion, we demonstrated that current procedural and perfusion approaches used for SAVR and TAVI determine pattern and degree of procedure-related inflammatory responses. The impact of the extracorporeal circulation technique seems to be substantial but not the sole determinant of the periprocedural inflammatory profile. Patients’ pre-treatment condition and the extent of myocardial trauma do also affect the inflammatory response. This may confound interpretation of biomarker profiles. Although, if possible, avoidance of any pro-inflammatory state prior to invasive medical procedures seems reasonable, our study could not establish an obvious link between the extent of the peri-procedural inflammatory response and clinical outcome parameters as IMC/ICU time or post-op hospital length of stay, incidence of new infection or 30-d mortality. Especially, in the population of TF-TAVI patients cardiac decompensation due to severe aortic stenosis led to IMC admission already in the pre-treatment period and thus to a prolonged IMC stay. In contrast, TA-TAVI patients exhibited the longest post-op hospital LOS, which can be partly interpreted as the result of the high incidence of SIRS in this population.

Within the range of current surgical and interventional treatment modalities for severe aortic valvular stenosis, TF-TAVI emerges as the approach with the smallest pro-inflammatory activation. According to laboratory (IL6, WBC) and clinical (SIRS) criteria, TA-TAVI has to be classified not as an interventional but as a true surgical procedure, with inflammatory biomarker profiles comparable to those found after SAVR.
